# Age, period, and cohort effects for future employment, sickness absence, and disability pension by occupational gender segregation: a population-based study of all employed people in a country (> 3 million)

**DOI:** 10.17269/s41997-019-00216-1

**Published:** 2019-05-14

**Authors:** Lena Gonäs, Anders Wikman, Kristina Alexanderson, Klas Gustafsson

**Affiliations:** Division of Insurance Medicine, Department of Clinical Neuroscience, Karolinska Institutet, SE-171 77 Stockholm, Sweden

**Keywords:** Occupation, Gender segregation, Employment, Public health, APC analysis, Sick leave, Professions, Ségrégation des sexes, Emploi, Santé publique, Analyse APC, Congé maladie

## Abstract

**Objectives:**

The occupational gender segregation of the labour market is very strong, both in Sweden and in North America. Nevertheless, there is little knowledge on how this is associated with employees’ future employment or morbidity. The objectives of this study were to explore age, period, and cohort effects on future employment and morbidity in terms of sickness absence (SA) or disability pension (DP) among women and men employed in numerically gender-segregated or gender-integrated occupations.

**Methods:**

Based on Swedish nationwide register data, three population-based cohorts of all people living in Sweden, with a registered occupation, and aged 20–56 years at inclusion in 1985 (*N* = 3,183,549), 1990 (*N* = 3,372,152), or 2003 (*N* = 3,565,579), respectively, were followed prospectively for 8 years each. First, descriptive statistics of employment and SA/DP at follow-up were calculated, related to level of gender segregation/integration of occupation at inclusion. Second, differences between birth cohorts (those born in 1929–1983, respectively) were estimated within each of the periods 1985–1993, 1990–1998, and 2003–2011, using mean polish analyses.

**Results:**

Women and men in gender-segregated occupations differed in relation to future employment rates and SA/DP. However, these differences decreased over time. Furthermore, the results show a birth cohort effect; those born in 1943–1956 remained in employment to a higher extent and also had lower rates of SA/DP than all other birth cohorts.

**Conclusion:**

Differences between people in the five categories of gender-segregated occupations decreased over time. Although age and period are important when explaining the outcome, also birth cohort effects have to be considered, both from a public and an occupational health perspective.

## Abstract (fr)

**Objectifs:**

La ségrégation professionnelle des sexes sur le marché du travail est très ancrée, tant en Suède qu’en Amérique du Nord. On en sait peu, toutefois, sur son association avec l’emploi ou la morbidité ultérieurs des employés. Dans cette étude, nous avons voulu explorer les effets de cette ségrégation sur l’emploi et la morbidité ultérieurs selon l’âge, la période et la cohorte, du point de vue de l’absentéisme pour maladie (AM) ou des rentes d’invalidité (RI) chez les femmes et les hommes travaillant dans des professions où il existe numériquement une ségrégation ou une intégration des sexes.

**Méthode:**

D’après les données du fichier national suédois, trois cohortes populationnelles de toutes les personnes vivant en Suède, ayant une profession inscrite et étant âgées de 20 à 56 ans au départ, en 1985 (*N* = 3,183,549), en 1990 (*N* = 3,372,152) ou en 2003 (*N* = 3,565,579), respectivement, ont été suivies prospectivement pendant huit années chacune. Premièrement, nous avons calculé les chiffres descriptifs pour l’emploi et l’AM/RI au suivi, en lien avec le niveau de ségrégation ou d’intégration des sexes de la profession au départ. Deuxièmement, nous avons estimé les différences entre les cohortes de naissance (les personnes nées entre 1929 et 1983, respectivement) pour chaque période, 1985–1993, 1990–1998 et 2003–2011, à l’aide d’analyses de polissage par la moyenne.

**Résultats:**

Les femmes et les hommes travaillant dans des professions où il existe une ségrégation des sexes présentaient des différences en ce qui a trait à leurs taux d’emploi et à leur AM/RI ultérieurs. Ces différences ont toutefois diminué dans le temps. De plus, les résultats indiquent un effet de la cohorte de naissance : les personnes nées entre 1943 et 1956 sont restées employées plus longtemps que dans les autres cohortes de naissance et ont présenté des taux d’AM/RI inférieurs.

**Conclusion:**

Les différences entre les personnes des cinq catégories de professions où il existe une ségrégation des sexes ont diminué dans le temps. Bien que l’âge et la période importent pour expliquer la résultante, la cohorte de naissance a aussi des effets dont il faut tenir compte, tant dans une perspective de santé publique que de santé au travail.

## Introduction

Health and life situations among people of different strata have been a major focus of public health research (Lancet 2018). Focus has to a large extent been on differences between people of, for example, lower and higher socio-economic status (SES), or between people of different ages, ethnicities, and sex, or living in urban versus rural areas. However, in most countries, there also is a high level of gender segregation of both paid and unpaid work (most occupations are numerically either female or male dominated). Nevertheless, associations between gender segregation and health have so far only been explored to a very limited extent, within public as well as occupational health. Studies show that paid work is good for your health, but there are gender-specific risks of leaving the labour market and of being on sickness absence (SA)/disability pension (DP) for the gender in minority in gender-segregated occupations (Gonas et al. 2018). In this study, we analyzed future health and employment outcomes considering the gender divisions of occupations and using Sweden as an example of a common international situation.

Alongside the growth of the female employment rate in the 1960s in Sweden, the gender segregation of the labour market increased (Gonäs and Tyrkkö 2015). This growth was supported by an increase in public daycare and parental-leave benefit systems (Mahon et al. 2012). Three decades later, women’s and men’s employment levels were almost equal, 79% for women and 83% for men (OECD 1996).

In a transatlantic comparison, this development was different in Canada and the United States. In 1990, the employment gap between women and men was still substantial (OECD 1996), even though the occupational gender segregation was similar to most EU countries (Bettio and Verashchagina 2009; Blau et al. 2013; Jarman et al. 2012).

During the last decades, the SA/DP rates have grown in most welfare states among both women and men (OECD 2010). The aim of this study was to explore to what extent women and men in gender-segregated and gender-integrated occupations, respectively, remained in employment over time as well as their risk of long-term SA/DP.

### Consequences of gender segregation—some theoretical starting points

Differences in what type of occupations women and men have may be due to discrimination or selectivity from the employers’ side (Stier and Yaish 2014). Women’s educational progress has increased their possibilities to apply for more qualified jobs, yet many studies show that women encounter a risk for being overqualified for the jobs they actually get (Goldin 2014). Some argue that to a great extent it is women’s choices that shape the segregation patterns. Occupations with short working hours or flexible working conditions can be a way to combine unpaid and paid work, although these choices may also lead to stressful working conditions that can explain women’s higher SA rates (Mastekaasa 2005; OECD 2010; Fagan and Burchell 2002). Results from some studies concerning the associations between gender segregation at work and SA/DP show that those in gender-integrated occupations have better health, operationalized as lower levels of SA, than those in gender-segregated occupations (Gonas et al. 2018; Kjellsson et al. 2014). They also show that a skewed gender balance in occupations or workplaces can have health consequences. This can affect the psychosocial working conditions, such as job demand, control, and social support (Mastekaasa 2005; Kroger 2017) and have consequences for the ability of individuals to continue in paid work and for future SA or DP risks (Mastekaasa 2005; Kroger 2017).

### Age, period, and cohort (APC) effects for future employment, SA, and DP by occupational gender segregation

Several aspects need to be considered in this type of analysis, such as the age of the women and men under study and the continually changing conditions taking place in the labour market, establishing cohort and period effects, all affecting the risk of unemployment, SA, and DP (Tukey 1977; Selvin 2004; Keyes et al. 2010). The risk of becoming unemployed is higher among younger people and among those with lower educational levels (Arnell 2006). Older people have higher risk of long-term unemployment than younger people, following redundancy or job loss (Farber 1999). SA and DP have clear age-related patterns, with higher levels among older people (Allebeck and Mastekaasa 2004).

Period effects reflect variation over time that affect different age groups simultaneously—through historical events and environmental factors such as labour market situation and shifts in economic crises. So far, for each period, a higher proportion in every age group are higher educated. The economic recession of the early 1990s led to lower employment levels and an increase in unemployment rates, as in the recession of 2008/2009 (Rubery and Rafferty 2013) and an increase in SA/DP in both Europe and North America (OECD 2010). Political measures to support equal opportunity developed early in Sweden. The Canadian policies have not been as comprehensive in these fields (Mahon et al. 2012). From having the male wage earner as the family provider, the roles have successively changed and moved toward the dual-career family, a trend in the Scandinavian countries that also is seen in a broad transnational perspective (Hobson 2002; Rubery 2015).

A birth cohort moves through life together and encounters the same historical situation at the same ages. Those entering the labour market in the early post-war period met expectations that were quite different concerning gender roles from those entering in the 1990s. Different birth cohorts carry different sets of values concerning gender roles and meet different labour market possibilities and problems, sometimes resulting in health problems and SA/DP (Allebeck and Mastekaasa 2004). They meet different labour market structures as they enter the labour market which also lead them into different labour market career paths (Granqvist and Persson 2005; Rubery and Rafferty 2013; Gonas et al. 2018).

### Research questions

1. Do future rates of employment and SA/DP differ between individuals in different categories of numerical occupational gender segregation?
2. Are period effects and variation over time a dominant force behind future rates of employment or SA/DP in different categories of numerical occupational gender segregation?
3. Does birth cohort membership influence the future rates of employment and SA/DP among individuals in different categories of numerical occupational gender segregation?

## Methods

This is an exploratory study, aiming at identifying possible mechanisms in a population, using the advanced APC analyses. We used annual information from Statistics Sweden’s Longitudinal Integration Database for Health Insurance and Labour Market Studies (LISA) concerning sex, age, type of occupation, emigration, income (from work, unemployment benefit, SA, DP, social security benefits, or student benefit), and number of days with SA/DP benefits. All people in Sweden are included in LISA for all years living here from 1990, including immigrants (however, not people seeking asylum in Sweden (e.g., refugees) before assessed as fulfilling the criteria for asylum). For 1985, we used information from the People and Housing Census of 1985 (FoB85).

### Study populations

Our aim was to study differences between birth cohorts (1929–1983), ages (20–56), and periods (1985–1993, 1990–1998, 2003–2011). Each individual was intended to be followed up 8 years after their occupation was registered, and we have followed the tradition in an age cohort analysis (Keyes et al. 2010). For each of these three periods, we included all people living in Sweden registered as working in an occupation from three population-based cohorts (in 1985 *N* = 3,183,549; in 1990 *N* = 3,372,152; and in 2003 *N* = 3,565,579). Those who had emigrated or died during the 8-year follow-up were excluded from period 1985–1993 (*n* = 124,819; 3.92%), period 1990–1998 (*n* = 149,675; 4.44%), and period 2003–2011 (*n* = 152,509; 4.27%).

### Exposure variables

The categorizations of level of occupational gender segregation of occupation in 1985, 1990, and 2003, respectively, were based on the gender distribution among all the occupations, at a three-digit level (112 occupations in 2003; 111 in 1985 and 1990). The Standard for Swedish Occupational Classification (SSYK-96) at the three-digit level was used (SSYK-96 closely follows the International Standard Classification of Occupations, ISCO-88 and the ISCO (COM), used in the statistical publications from the European Union (Bettio and Verashchagina 2009). Information about peoples’ occupations at inclusion in respective population cohort was for 1985 obtained from FoB85 and for 1990 and 2003 from LISA. The reason for this selection of years was that LISA had no occupational information for the years 1991–2002. Occupations were grouped into five categories according to whether they were numerically dominated by women or men. The classifications used were similar to other studies (Kumlin 2010): extremely female dominated (≥ 90% women), e.g., office secretaries, nursing, and midwifery professionals; female dominated (≥ 60– < 90% women), e.g., personal care, helpers, and cleaners; gender integrated (≥ 40– < 60% women), e.g., public service administrative professionals, secondary education teaching professionals; male dominated (≥ 10– < 40% women), e.g., finance and sales professionals, directors, and chief executives; extremely male dominated (< 10% women), e.g., transport workers, building workers (Gonäs et al. 2018).

### Outcome variables

The individuals were followed up regarding their labour market position or main source of income at 8 years after inclusion, that is in 1993, 1998, and 2011, respectively. An 8-year follow-up was used because this was the only alternative given by the data to get a comparable follow-up period for all three periods.

The employment situation at follow-up was classified according to a way that Statistics Sweden had classified people as being employed or not employed, based on the size of their annual income from work. This categorization was refined by using complementary data concerning different social security benefits (parental leave, student benefit, unemployment, SA, DP, old-age pension) (Wikman et al. 2012).

According to this classification, each individual was assigned to one of the following categories: (a) employment (including self-employed), (b) parental leave benefit, (c) student benefit, (d) SA, (e) DP, (f) unemployment benefit, (g) social assistance benefit, (h) unknown (no type of registered income or benefit), (i) old-age pension. These nine categories were then combined into two dichotomized outcome variables. For description of the subgroups and dichotomization, see Gonäs et al. 2018.

The first outcome concerned employment status and was dichotomized as:

- *Employed*, including self-employed (a) and those on parental leave (b)
- *Not employed*, i.e., (c) those with student benefit, (d) SA, (e) DP, (f) unemployment benefit, (g) social assistance benefit, (h) unknown, no type of registered income, and (i) old-age pension.

The second outcome concerned sickness absence benefits, dichotomized as:

- *Sickness absence or disability pension*, i.e., categories (d) and (e)
- *All other categories*, i.e., (a) employed (including self-employed), (b) parental leave, (c) student benefit, (f) unemployment benefit, (g) social assistance benefit, (h) unknown, no type of registered income, and (i) old-age pension.

### Data analyses

First, rates of employment or levels of SA/DP at 8-year follow-up were calculated for each period and each occupational group, stratified by gender.

Second, rates of employment and rates of SA/DP were calculated for the five occupational categories of gender segregation, together with age, and period (presented in two types of diagrams).

Finally, an APC analysis was conducted regarding age (A), period (P), and cohort (C) effects. For this, we used mean polish approach (Tukey 1977; Selvin 2004; Keyes et al. 2010)*.* This approach evaluates the remaining covariation (residuals) between birth cohorts and outcomes when covariations in age and period have been considered and excluded. Age (A) was retained as a continuous variable (28–64 years). The period (P) refers to the three 8-year periods: 1985–1993, 1990–1998, and 2003–2011, respectively. Cohort (C) was based on birth cohorts, 1929–1983.

The residuals from the APC analysis were then regressed on birth cohort membership using univariate logistic regression analysis with the dependent variables “employment” respectively “SA/DP”.

For different birth cohorts, the remaining differences in employment, SA, and DP were calculated when covariations in age and period were eliminated.

The analysis quantifies the influence of observed birth cohort effects on employment respective SA/DP, using the average of residuals in each period as reference points. These average values were assumed to be a constant component associated with each birth cohort. A value greater than zero indicates higher than average influence and less than zero indicates lower than average influence from age/period effects on employment rate and SA or DP rate. For estimated birth cohort effects, the results of the univariate logistic regression are presented as odds ratios (OR) with 95% confidence intervals (CI).

All data used in the study were de-identified by Statistics Sweden before being made available to the research team. The project was approved by the Regional Ethical Review Board in Stockholm, Sweden (no. 2007/762-31 2012/863-32).

## Result

The employment rate at the 8-year follow-up increased for most occupational groups with the highest rate in the last period. Two exceptions were the extremely female-dominated and female-dominated groups with no or very small changes between the three periods (Table 1).

**Table 1:** Frequency and percentages of status of employment, sickness absence/disability pension (SA/DP), and all other categories at 8-year follow-up (in 1993, 1998, 2011), among the women and men who at inclusion in the three different population-based cohorts worked in the five respective occupational gender-segregation categories (in 1985, 1990, 2003)

	Extremely female dominated		Female dominated		Gender integrated		Male dominated		Extremely male dominated	
	*n*	%	*n*	%	*n*	%	*n*	%	*n*	%
Women										
Employed 1993	150,297	87.6	729,186	78.1	125,538	80.3	135,016	70.2	12,086	64.6
Employed 1998	164,284	86.2	736,556	75.4	151,203	81.2	155,764	71.9	14,514	66.3
Employed 2011	164,104	87.2	856,975	80.6	187,513	87.0	188,116	81.3	13,509	76.1
SA/DP 1993	10,034	5.8	110,833	11.9	14,169	9.1	30,432	15.8	3615	19.3
SA/DP 1998	14,584	7.7	118,346	12.1	15,856	8.5	29,418	13.6	3564	16.3
SA/DP 2011	17,379	9.2	114,282	10.7	14,891	6.9	23,711	10.2	2270	12.8
All other 1993^a^	11,195	6.5	94,065	10.1	16,658	10.6	26,829	14.0	3022	16.1
All other 1998^a^	11,715	6.1	122,248	12.5	19,115	10.3	31,481	14.5	3817	17.4
All other 2011^a^	6794	3.6	92,447	8.7	13,196	6.1	19,633	8.5	1981	11.1
All women 1993	171,526	100	934,084	100	156,365	100	192,277	100	18,723	100
All women 1998	190,583	100	977,150	100	186,174	100	216,663	100	21,895	100
All women 2011	188,277	100	1,063,704	100	215,600	100	231,460	100	17,760	100
Men										
Employed 1993	8583	88.6	123,552	76.9	197,044	83.9	549,274	78.8	347,551	71.8
Employed 1998	9507	88.8	130,011	76.4	208,890	86.1	579,460	82.5	382,520	75.9
Employed 2011	15,362	89.1	242,989	82.2	224,423	89.4	653,937	87.6	329,940	85.6
SA/DP 1993	402	4.2	13,563	8.4	12,776	5.4	58,203	8.4	53,616	11.1
SA/DP 1998	539	5.0	13,669	8.0	12,499	5.2	49,932	7.1	47,220	9.4
SA/DP 2011	896	5.2	17,236	5.8	10,002	4.0	35,990	4.8	23,065	6.0
All other 1993^a^	697	7.2	23,647	14.7	24,976	10.7	89,137	12.8	82,734	17.1
All other 1998^a^	663	6.2	26,543	15.6	21,159	8.7	73,238	10.4	74,162	14.7
All other 2011^a^	980	5.7	35,530	12.0	16,572	6.6	56,955	7.6	32,392	8.4
All men 1993	9682	100	160,762	100	234,796	100	696,614	100	483,901	100
All men 1998	10,709	100	170,223	100	242,548	100	702,630	100	503,902	100
All men 2011	17,238	100	295,755	100	250,997	100	746,882	100	385,397	100

^a^All other categories, i.e., (c) student benefit, (f) unemployment benefit, (g) social assistance benefit, (h) unknown (no type of registered income or benefit), (i) old-age pension. Not included: those who had emigrated or died during the follow-up

On the other hand, the proportion of women and men with SA/DP increased in the categories extremely female dominated and female dominated but decreased in all other occupational gender-segregation categories.

**Fig. 1:**
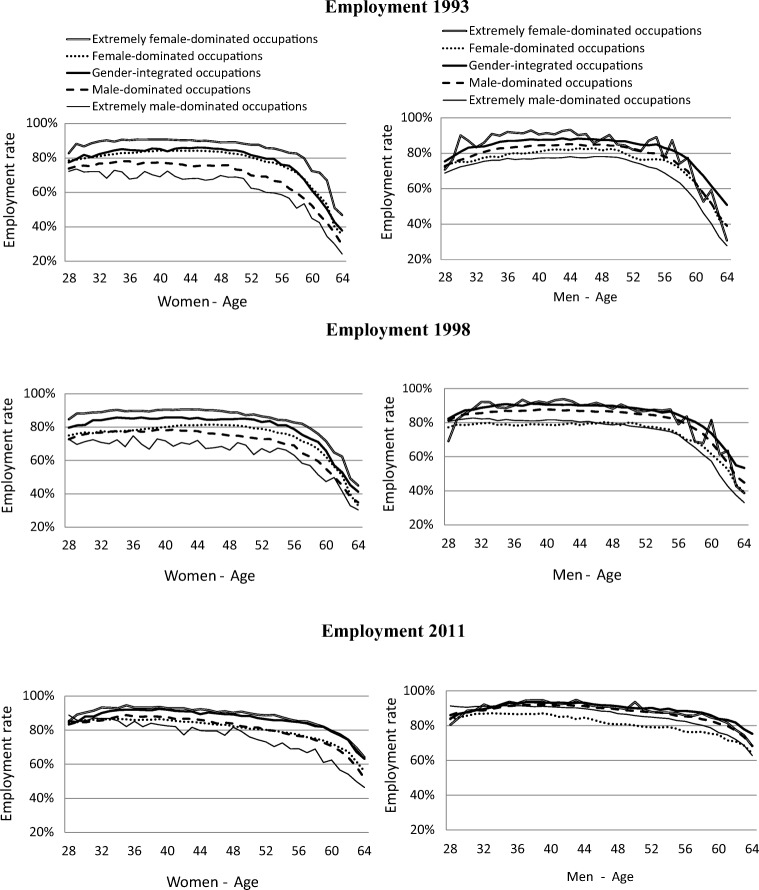
Prevalence of employment rate for age (28–64 years), and period time (1993, 1998, 2011) by occupation gender-segregation categories stratified by gender

Figure 1 describes the same data but now with age considered. The figure shows that employment rates declined with age group in all occupational gender-segregation categories among both women and men between 1993 and 2011. Women had the same inverted U-shaped age-related employment rate curves as men, common in the Nordic welfare states (Rubery et al. 1999). The employment rates at follow-up among older women (age 60) increased, from 45–75% in the period 1985–1993 to 60–80% in the period 2003–2011. Among older men, the corresponding figures were 50–70% and 75–85%. The differences in employment rates between the categories of occupational gender segregation decreased with period for both women and men. However, the differences in employment rates were less pronounced among men compared with among women in all periods.

**Fig. 2:**
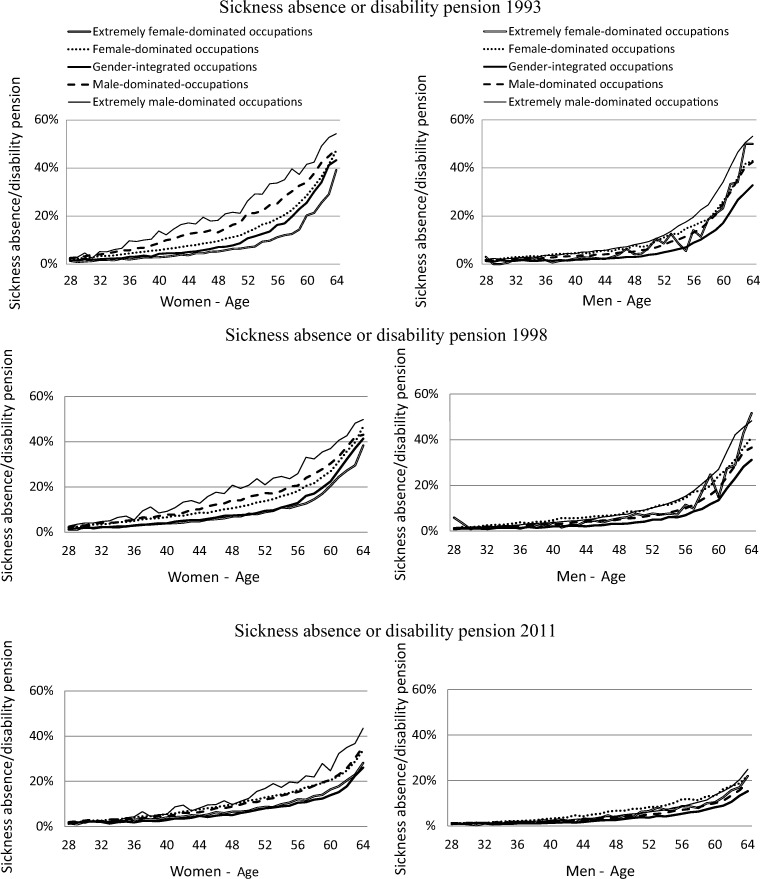
Prevalence of sickness absence or disability pension rate for age (28–64 years), and period time (1993, 1998, 2011) by occupation gender-segregation categories stratified by gender

Figure 2 shows that SA/DP rates increased with age in all five categories of occupational gender segregation, among both women and men. These increases were more pronounced in the earlier periods. Figure 2 also shows that the differences in SA/DP rates between people initially working in the different gender-segregated occupational categories decreased over the years.

The SA/DP rates among women at age 64 were approximately 55% in the extremely male dominated and 40% in the extremely female-dominated occupational categories in the period 1985–1993. The corresponding figures for period 1990–1998 were relatively unchanged, and for the period 2003–2011, 45% and 35%, respectively. Among men in period 1985–1993, the SA/DP rates at follow-up among those then aged 64 were about 55% for those initially in the extremely male-dominated occupations and 50% among those initially in the extremely female-dominated occupations. The corresponding figures for period 1990–1998 were similar and for period 2003–2011, 25% and 22%, respectively, at follow-up**.**

**Fig. 3:**
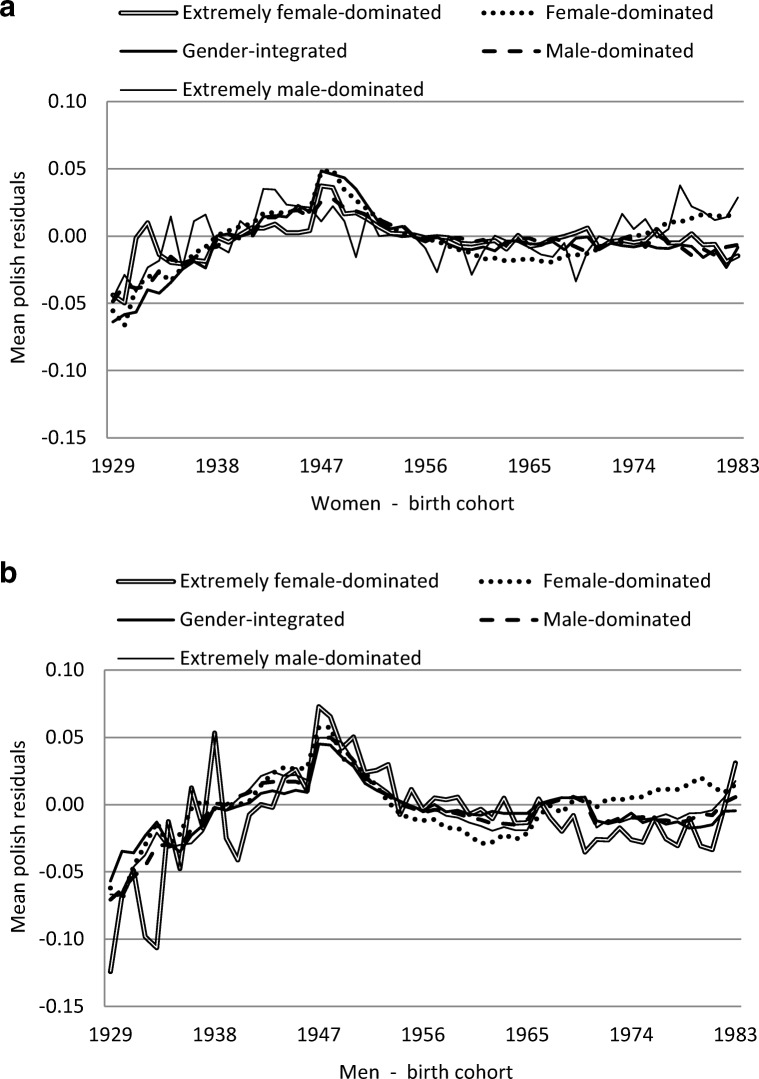
**a***Women* and **b***men* average of the estimate residuals quantifying a specific birth cohort effect from the employment data related to the five occupational gender-segregation categories

Figure 3 a and b show that the residual values on employment raised continuously for the birth cohorts born 1929–1950, then fell at year 1960 for both women and men. This indicated a more favourable situation for the birth cohorts born around 1943 to 1956 regarding their employment rate. After 1960, the residual values contained only small effects. There were minor differences between women and men, where the pattern for men was more pronounced with a variation between the birth cohorts specifically for those in occupations belonging to the extremely female-dominated category.

Table 2 shows the descriptive results of the univariate logistic regression analysis. Compared with the reference category of birth cohort 1929–1942, those in birth cohorts 1943–1956 were significantly more likely to be employed. Further, those in later birth cohorts were significantly less likely to be employed.

**Table 2:** Univariate logistic regression (residuals from mean polish approach): birth cohort effects on employment and sickness absence or disability pension (SA/DP) in Sweden 1993–2011

	Extremely female dominated			Female dominated			Gender integrated			Male dominated			Extremely male dominated		
	OR	CI 95%		OR	CI 95%		OR	CI 95%		OR	CI 95%		OR	CI 95%	
Employment															
Women															
Birth cohort (1929–1983)															
1929–1942 reference	1			1			1			1			1		
1943–1956	*14.28*	13.97	14.60	*4.39*	4.35	4.43	*7.39*	7.21	7.57	*19.96*	19.96	18.89	*2.19*	2.04	2.35
1957–1969	*0.05*	0.05	0.06	na			*0.10*	0.10	1.10	*0.56*	0.55	0.57	*0.11*	0.10	0.12
1970–1983	*0.47*	0.46	0.48	*1.28*	1.12	1.14	*0.10*	0.09	0.10	*0.34*	0.33	0.34	*1.54*	1.43	1.67
Men															
Birth cohort (1929–1983)															
1929–1942 reference	1			1			1			1			1		
1943–1956	*16.67*	14.56	19.22	*6.95*	6.78	7.12	*16.58*	16.28	16.88	*4.07*	4.03	4.12	*18.48*	18.19	18.77
1957–1969	*2.42*	2.10	2.79	*0.25*	0.24	0.25	*1.33*	1.30	1.35	*0.16*	0.16	0.16	*0.34*	0.34	0.35
1970–1983	*0.08*	0.06	0.10	na			*0.41*	0.40	0.42	*0.19*	0.18	0.19	*0.51*	0.50	0.51
Sickness absence or disability pension (SA/DP)															
Women															
Birth cohort (1929–1983)															
1929–1945 reference	1			1			1			1			1		
1946–1962	*0.44*	0.43	0.46	*0.09*	0.09	0.09	*0.05*	0.05	0.06	*0.01*	0.01	0.01	*0.05*	0.05	0.06
1963–1983	na			na			1.01	0.94	1.08	*0.21*	0.20	0.22	*0.25*	0.22	0.29
Men															
Birth cohort (1929–1983)															
1929–1945 reference	1			1			1			1			1		
1946–1962	*0.21*	0.16	0.26	*0.04*	0.04	0.04	*0.08*	0.07	0.08	*0.01*	0.02	0.02	na		
1963–1983	na			na			na			*0.48*	0.46	0.50	*0.81*	0.77	0.85

Italics = statistically significant at the *p* < 0.05 level

*na* not available, was not estimated

**Fig. 4:**
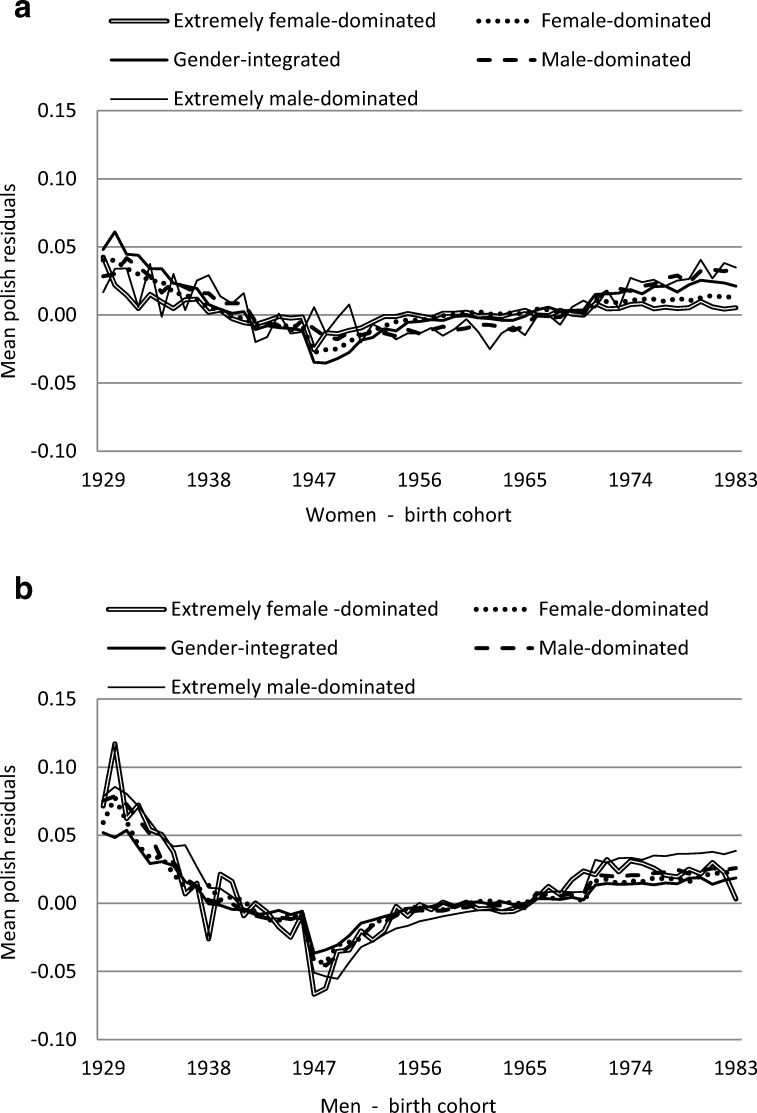
**a***Women* and **b***men* average of the estimate residuals quantifying a specific birth cohort effect from the sickness absence or disability pension data related to the five occupational gender-segregation categories

Figure 4 a and b show that the residual values decreased for the birth cohorts born between 1929 and 1950 regarding SA/DP for both women and men. After the year 1960, the residual values contained only small or essentially random effect. This indicated again a more favourable situation for the birth cohorts born 1943–1956 with lower risk of SA/DP. However, again, there was a minor difference in terms of gender. Significant birth cohort effects were observed (Table 2). Compared with the reference category of birth cohorts 1929–1945, those in birth cohorts 1946–1962 were significantly less likely to have SA or DP.

## Discussion

The aim of this population-based prospective cohort study was to explore future employment and long-term SA or DP among women and men in different categories of occupational gender segregation, using large population-based cohorts.

The results in Table 1 show large differences in the employment levels 8 years after inclusion between women who worked in different categories of occupational gender segregation. Women initially in extremely male-dominated occupations in 1985 had an employment level 8 years later of 64.6%, and women in extremely female-dominated occupations 87.6%. This difference is remarkable compared with what is the case for men in the same situations, 71.8% versus 88.6%. The results are partly in accordance with other studies that claim that the gender in minority can have a vulnerable position at work (Kroger 2017). Results from a Swedish study (Kjellsson et al. 2014) show that a skewed gender balance in workplace could involve poor psychosocial working conditions.

For both women and men, the highest employment rate in the period 1985–1993 was in the extremely female-dominated category; this was also the case in the other two periods. The largest increases in the employment rates, for both women and men, in the periods were in the extremely male-dominated occupations. At the same time, the differences between the three periods decreased between the different occupational gender categories concerning later employment and later SA/DP (Figs. 1 and 2).

Over time, the chance to remain in employment seemed to be higher among those initially being in minority in a gender-segregated occupation. This trend was more pronounced among women than among men, indicating that there might have been some improvements for women working in male-dominated occupations. Other interpretations could be that women entered with less difficulty into male-dominated occupations and that they have higher qualifications today which give them a stronger labour market position (Bettio and Verashchagina 2009), or to a higher degree moved to other types of occupations. A continuously more equal mix of women and men may also have changed the working condition. Although the Swedish labour market is still gender segregated, the trend seems to be toward a more equal balance (Bettio and Verashchagina 2009; Kumlin 2010). Women may have reached better jobs, better working conditions, and employment security (Kjellsson et al. 2014). Such tendencies toward a more equalized labour market could be less visible in general statistics, due to the complex mix of involved factors. Our results give a more detailed description of some aspects of these changes than more aggregated information manages to provide, e.g., through Gini-index or ID-index, where different trends can cancel each other out.

In the next type of calculations, we estimated the differences between the five categories of occupational gender segregation when effects according to age and periods were excluded (Figs. 3 and 4). Still, some differences remained. It appeared that people born in the middle or last part of the 1940s had better chances to remain as employed later in their lives and with lower risk of future SA/DP. All birth cohorts had a positive development regarding being in employment and a low level of SA/DP, most pronounced for people born in the 1940s and early 1950s. This latter group may have had, in Sweden as well as in some other countries (Putnam 2015), a more favourable situation when they as young people entered the labour market. They may have had less difficulties in finding adequate jobs due to better education opportunities (SCB 2016) and better career possibilities than previous cohorts. Later birth cohorts, on the other hand, may have had less favourable opportunities due to higher unemployment rates and greater difficulties in finding permanent employment. One may assume some lasting effects of this dramatic labour market situation in the later working life for these individuals. Such birth cohort effects need to be studied further from a broad public health perspective.

Strengths of this study were that all employed in a whole country could be included; that is, no selection bias, the use of high-quality register data (no self-reports), no loss to follow-up, the large cohorts allowing for subgroup analyses, and the long follow-up. SA and DP are good measures of consequences of morbidity, in terms of work incapacity; that is, not being able to support yourself from work due to disease or injury, a measure increasingly used in public health studies. The associations between gender segregation of the labour market and health outcomes need to be studied further.

### Conclusion

The initial differences between categories of occupational gender segregation regarding future employment rates and SA and DP decreased between the three studied periods among both women and men. Over time, it seems to become easier to remain in employment among those initially working in a gender-segregated occupation, among both women and men. The development of SA and DP differed in that respect. We found a birth cohort effect for both women and men, where those born in 1946–1956 had a higher chance of remaining in employment and lower risk of future long-term SA and DP than those in younger birth cohorts. From a public health perspective, these birth cohort effects need to be studied further.
